# Efficacy of 3rd generation TKI in patients with EGFR mutation lung adenocarcinoma with bone metastases: A review of 3 case reports and literature

**DOI:** 10.1097/MD.0000000000034545

**Published:** 2023-08-25

**Authors:** Qiang Guo, Weiqiang Feng, Sheng Hu, Jiayue Ye, Silin Wang, Lang Su, Yang Zhang, Deyuan Zhang, Wenxiong Zhang, Jianjun Xu, Yiping Wei

**Affiliations:** a Department of Thoracic Surgery, The Second Affiliated Hospital of Nanchang University, Nanchang, China.

**Keywords:** bone metastases, EGFR 19del mutation, lung adenocarcinoma, osimertinib, third-generation EGFR-TKIs

## Abstract

**Rationale:**

With the advancement of targeted therapies, epidermal growth factor receptor tyrosine kinase inhibitors have become the preferred initial treatment for patients with advanced epidermal growth factor receptor (EGFR)-mutated non-small cell lung cancer. Osimertinib, a third-generation epidermal growth factor receptor tyrosine kinase inhibitor, is effective against exon 19 and 21 mutations as well as the T790M mutation. It has been approved by both the food and drug administration and European Medicines Agency for the treatment of non-small cell lung cancer patients with locally advanced or metastatic EGFR-mutated tumors, including those who have acquired T790M mutations.

**Patient concerns:**

To evaluate the effectiveness of osimertinib in treating patients with EGFR-mutated advanced lung adenocarcinoma and bone metastases, we present the treatment outcomes of 3 patients with EGFR 19 deletion-mutated advanced lung adenocarcinoma and bone metastases who received osimertinib treatment in recent years. All 3 cases involved elderly female patients, aged 62, 62, and 54, respectively.

**Diagnoses:**

All 3 cases exhibited a diagnosis of pulmonary adenocarcinoma accompanied by osseous metastases, with genetic testing revealing the presence of an EGFR 19del mutation.

**Interventions:**

In the first case, following 17 months of gefitinib therapy, disease progression prompted a switch to osimertinib treatment. In the second case, bone metastases were detected after 20 months of pemetrexed-carboplatin chemotherapy, leading to a transition to osimertinib therapy. In the third case, after 11 months of erlotinib treatment, bone metastases were identified. Subsequent interventions, including radiation therapy, pemetrexed-carboplatin chemotherapy, pemetrexed-bevacizumab maintenance therapy, and docetaxel chemotherapy, failed to arrest the progression of bone metastases. As a result, a combination of osimertinib and anlotinib targeted therapy was administered.

**Outcomes:**

All 3 patients experienced relatively good and favorable survival outcomes, with a progression-free survival of 22.7 months, 12 months, and 17.7 months, respectively.

**Lessons:**

These cases suggest that osimertinib is a promising treatment option for patients with EGFR 19 deletion-mutated lung adenocarcinoma and bone metastases, although further clinical studies are needed to confirm its efficacy.

## 1. Introduction

Currently, both domestic and international studies indicate that lung cancer is the most commonly diagnosed cancer worldwide.^[1]^ Among all the lung cancers, adenocarcinoma accounted for 40%. In Asian non-small cell lung cancer (NSCLC) patients, approximately 1 to 3rd have epidermal growth factor receptor (EGFR) mutations. For these patients, epidermal growth factor receptor tyrosine kinase inhibitors (EGFR-TKIs) have been shown to be more effective and offer longer progression-free survival (PFS).^[2]^ Currently, EGFR-TKIs have emerged as the first-line treatment option for patients with advanced NSCLC harboring EGFR mutations.^[3,4]^ The third-generation EGFR-TKIs exhibit activity against mutations in exon 19 and 21, as well as the T790M mutation.^[5]^ Among these third-generation EGFR-TKIs, osimertinib (ADZ9291) was the first to receive approval from both the food and drug administration and the European medicines agency in November 2015 and February 2016, respectively. It is indicated for the treatment of patients with quasi-metastatic EGFR mutation and acquired EGFR T790M mutation-positive NSCLC. However, there is limited research on the efficacy of osimertinib in treating patients with advanced EGFR-mutated lung adenocarcinoma with bone metastases, and its efficacy and safety remain unclear. In this report, we present the treatment course of 3 patients with advanced EGFR 19 deletion-mutated lung adenocarcinoma with bone metastases who were treated with osimertinib.

## 2. Case presentation

### 2.1. Case 1

**Figure 1.**
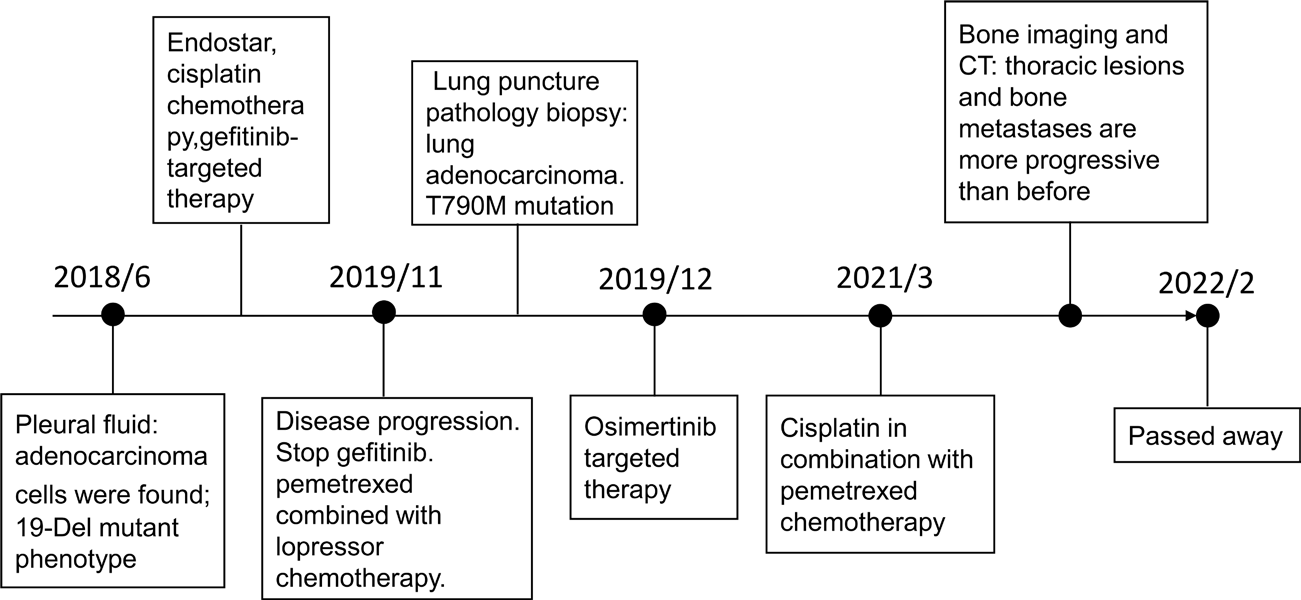
The process of diagnosis, treatment, and outcome of case 1.

A 62-year-old woman presented with a 3-month history of chest distress and shortness of breath. On physical examination, breath sounds were absent in the left lung and diminished in the right lung. Ultrasound of the pleural fluid revealed a large effusion on the left side of the chest. Thoracentesis showed elevated levels of carcinoembryonic antigen and carbohydrate antigen 125, and the pathological diagnosis of the pleural fluid exfoliated cytology specimen was adenocarcinoma cells of pulmonary origin. Combined testing revealed a 19Del mutant phenotype. The patient was diagnosed with lung adenocarcinoma and malignant pleural effusion. Treatment with gefitinib-targeted therapy and chest-injection cisplatin chemotherapy started in June 2018. Tumor angiogenesis was inhibited with endostar to inhibit tumor cell growth. The patient’s pleural effusion was slightly reduced after treatment. One year later, repeat chest computed tomography (CT) scans showed higher pleural effusion than before, suggesting disease progression due to patient resistance to gefitinib. In November 2019, osimertinib was used as a targeted therapy after lung puncture pathology biopsy and next generation sequencing genetic testing revealed T790M mutation. The patient had a PFS of 22.7 months and an overall survival (OS) of 26.2 months. On October 7, 2021, whole body bone imaging revealed increased bone metastases, and the patient passed away on February 20, 2022, with persistent bone pain. (Fig. 1)

### 2.2. Case 2

The 62-year-old woman in this case presented with a cough that had persisted for more than 2 months. Chest CT showed bronchial stenosis and occlusion in the dorsal segment of the lower lobe of the left lung with occupancy in the lower lobe of the left lung, which raised suspicion for peripheral type lung cancer. She underwent thoracoscopic lobectomy, which confirmed the diagnosis of adenoidal alveolar lung cancer in the left lower lung. Postoperative staging was pT3N1M0 stage III, with metastasis to lymph nodes from group 8, 9, and 11.

**Figure 2.**
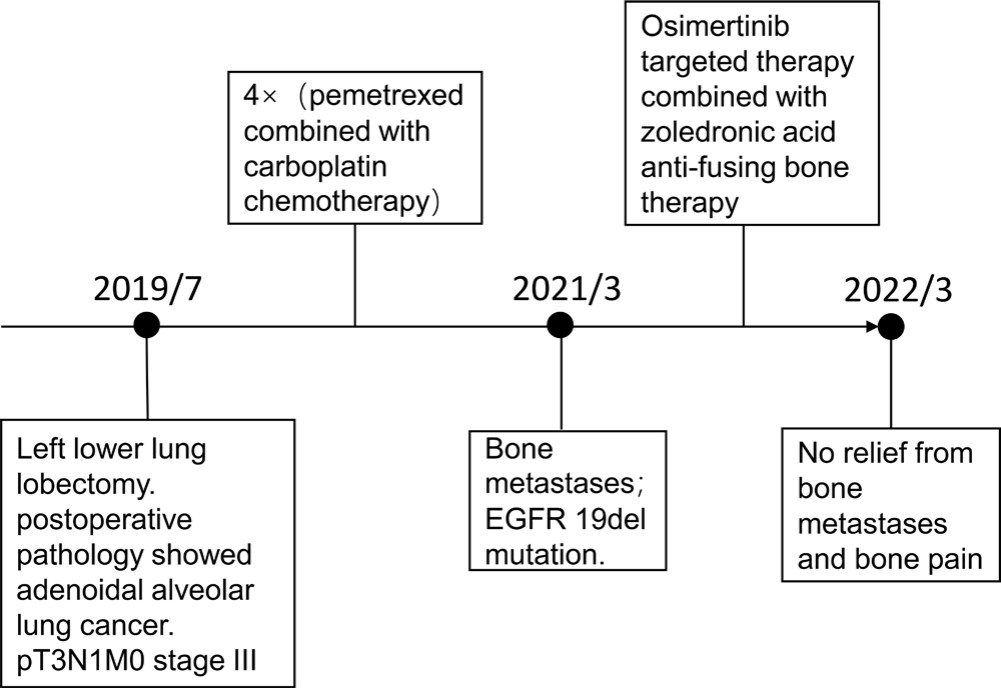
The process of diagnosis, treatment, and outcome of case 2.

The patient underwent 4 cycles of pemetrexed combined with carboplatin chemotherapy starting on August 7, 2019. In March 2021, bone metastases were suspected, and genetic testing showed EGFR 19del mutation. She was started on oral osimertinib targeted therapy and zoledronic acid anti-fusing bone therapy. CT on March 13, 2022, showed no significant change in segmental solid lesions in both lungs compared to before, and the right breast lesion and increased density of T12 and L2 vertebrae were approximately similar to before. However, the patient reported that the bone pain in the back and hip had not been relieved. (Fig. 2)

### 2.3. Case 3

**Figure 3.**
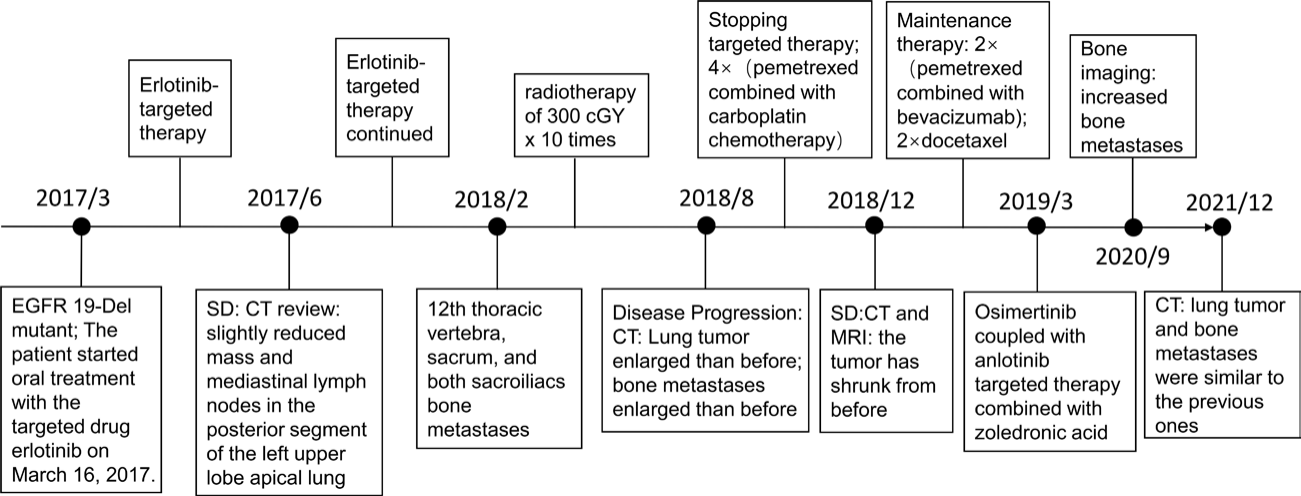
The process of diagnosis, treatment, and outcome of case 3.

The patient, a 54-year-old woman, was diagnosed with adenocarcinoma of the left lung in March 2017. Gene testing showed a 19Del mutation. A small speckle-like enhancement was found in the right temporal lobe, indicating metastasis. Treatment with erlotinib, a targeted drug, was started, and a review CT showed slightly reduced mass and mediastinal lymph nodes. However, small nodules in the left lung and right lower lobe remained, and scattered small ischemic foci were found in the brain. The patient’s condition progressed, and bone metastases were detected in February 2018. Radiotherapy was started, and the disease continued to progress, leading to the suspension of targeted therapy and the start of chemotherapy with pemetrexed and carboplatin. The tumor shrunk after 4 cycles of chemotherapy, and maintenance therapy with pemetrexed and bevacizumab was administered. However, the patient’s condition worsened again, and docetaxel was found to be ineffective. The patient then started osimertinib coupled with anlotinib targeted therapy combined with zoledronic acid anti-osteolytic therapy. Bone imaging showed new lesions in the left 4th and 6th ribs, and CT on December 15, 2021 showed no significant change in the lung lesion or bone metastases. The patient had a PFS of 17.7 months. (Fig. 3)

## 3. Discussion

This report outlines the treatment of 3 patients with advanced EGFR 19Del mutated lung adenocarcinoma and bone metastases using osimertinib. The first patient had initially been treated with gefitinib for 17 months, but became resistant to EGFR T790M mutation. After 22 months of osimertinib targeted therapy combined with chemotherapy, the lung tumor showed no significant change, and bone metastases progressed. The second patient underwent a lobectomy and 4 cycles of chemotherapy before bone metastases and EGFR 19Del mutation were detected after 8 months. Treatment was then changed to osimertinib combined with zoledronic acid, resulting in no significant change in lung tumor and bone metastases after 1 year. The third patient had undergone repeated chemotherapy, radiotherapy, and first-generation EGFR-TKIs over a 2-year period before disease progression. Targeted therapy with osimertinib in combination with anlotinib was then started, but after 18 months, bone metastases increased. All 3 patients with EGFR 19Del mutated bone metastatic lung adenocarcinoma had a relatively long stabilization period (12–22.7 months) of bone metastases after treatment with osimertinib. The data suggests that osimertinib is effective in treating bone metastases in patients with EGFR 19Del mutated lung adenocarcinoma.

Osimertinib has become a new standard of care for first-line treatment of patients with advanced NSCLC that have mutations in the EGFR gene. It has shown efficacy against the most common EGFR mutations, including exon 19 and 21 mutations, as well as the T790M resistance mutation that often arises during treatment with first-generation EGFR inhibitors.^[5]^ Many studies have demonstrated that osimertinib not only effectively controls the primary tumor, but also has greater penetration and brain exposure than other EGFR tyrosine kinase inhibitors such as gefitinib, erlotinib, or afatinib,^[6]^ resulting in sustained tumor remission in metastases.^[7]^ However, there is currently limited research on the efficacy of osimertinib in treating bone metastases from lung cancer, and there are currently no established systemic treatment regimens specifically for bone metastases. A real-world study conducted in China by Tang et al^[3]^ reported that bone metastases were a significant predictor of lower objective response rate and poorer PFS/OS with osimertinib treatment, suggesting that osimertinib may have a reduced efficacy in treating bone metastases. However, further research is needed to fully understand the potential limitations of osimertinib in treating bone metastases. Similar results were reported by Gu et al^[8]^ (2017): osimertinib was effective in controlling the primary disease in a 65-year-old patient with gefitinib-resistant T790M mutation lung adenocarcinoma, but was not effective in treating bone metastases (which progressed after 2 months of osimertinib use). These results differ from ours, which may be influenced by differences in study methods, study populations, and sample sizes. However, several studies have confirmed that osimertinib is a promising clinical option for patients with NSCLC with bone metastases. Takashi et al^[9]^ demonstrated that osimertinib regressed bone tumors in mice with EGFR-mutant lung adenocarcinoma and bone metastases, resulting in improved survival and bone remodeling in the mice. Similarly, Ying et al^[10]^ and Yoichi et al^[11]^ reported that patients treated with osimertinib experienced regression of bone metastases and longer-term survival rates. These findings align with those of our study, in which patients with bone metastases from lung adenocarcinoma with EGFR 19del mutation had longer-term control of both primary tumors and bone metastases, with PFS of 18 and 22 months and even longer OS after targeted therapy with osimertinib, following different treatments such as chemotherapy, radiotherapy, and surgery.

Behind breast and prostate cancers, NSCLC is the third most common cause of bone metastases.^[12]^ Metastatic bone tumors often occur in patients who experience bone-related events, particularly spinal metastases. These tumors can cause pain, instability, and vertebral collapse, leading to surgical emergencies with significant public health and economic consequences. The impact on patients quality of life can be profound.^[13]^ The efficacy of osimertinib in NSCLC patients with bone metastases remains controversial.^[9]^ The cases in this report remind us that osimertinib may be a promising clinical option for patients with EGFR 19del mutation with lung cancer with bone metastases, and future prospective studies with large sample sizes are needed to further validate the efficacy of osimertinib in patients with bone metastases from NSCLC.

## 4. Conclusion

Osimertinib shows promise as a clinical option for patients with EGFR 19del mutated lung adenocarcinoma and bone metastases, but further clinical studies are necessary to demonstrate its efficacy.

## Acknowledgments

We would like to gratefully acknowledge to the National Natural Science Foundation of China (81860379 and 82160410) and Jiangxi Provincial Science and Technology Department Key R&D Program [grant number 20223BBG71009] for supporting this study.

## Author contributions

**Conceptualization:** Qiang Guo, Weiqiang Feng, Yiping Wei.

**Data curation:** Qiang Guo, Weiqiang Feng, Silin Wang, Wenxiong Zhang.

**Formal analysis:** Qiang Guo, Sheng Hu.

**Funding acquisition:** Yiping Wei.

**Investigation:** Jiayue Ye, Lang Su, Yang Zhang, Deyuan Zhang.

**Project administration:** Yiping Wei.

**Resources:** Qiang Guo, Weiqiang Feng.

**Supervision:** Yiping Wei.

**Visualization:** Qiang Guo.

**Writing – original draft:** Qiang Guo, Weiqiang Feng.

**Writing – review & editing:** Qiang Guo, Sheng Hu, Silin Wang, Wenxiong Zhang, Jianjun Xu, Yiping Wei.

## References

[1] Vieira AR Abar L Vingeliene S. Fruits, vegetables and lung cancer risk: a systematic review and meta-analysis. Ann Oncol. 2016;27:81–96. 26371287 10.1093/annonc/mdv381

[2] Kobayashi K Kaira K Iemura H. Combination of immune check inhibitor and immunomodulatory arabinomannan extracted from mycobacterium tuberculosis: a case report. Mol Clin Oncol. 2021;15:227. 34631053 10.3892/mco.2021.2390 PMC8461616

[3] Tang X Li Y Qian WL. A comprehensive prognostic analysis of Osimertinib treatment in advanced non-small cell lung cancer patients with acquired EGFR-T790M mutation: a real-world study. J Cancer Res Clin Oncol. 2021. 10.1007/s00432-021-03797-y PMC11801141 34536138

[4] Yu HA Arcila ME Rekhtman N. Analysis of tumor specimens at the time of acquired resistance to EGFR-TKI therapy in 155 patients with EGFR-mutant lung cancers. Clin Cancer Res. 2013;19:2240–7. 23470965 10.1158/1078-0432.CCR-12-2246 PMC3630270

[5] Remon J Steuer CE Ramalingam SS. Osimertinib and other third-generation EGFR TKI in EGFR-mutant NSCLC patients. Ann Oncol. 2018;29(suppl_1):ii20–i27. 10.1093/annonc/mdx704 29462255

[6] Ballard P Yates JW Yang Z. Preclinical comparison of Osimertinib with other EGFR-TKIs in EGFR-Mutant NSCLC brain metastases models, and early evidence of clinical brain metastases activity. Clin Cancer Res. 2016;22:5130–40. 27435396 10.1158/1078-0432.CCR-16-0399

[7] Kishikawa T Kasai T Okada M. Osimertinib, a third-generation EGFR tyrosine kinase inhibitor: a retrospective multicenter study of its real-world efficacy and safety in advanced/recurrent non-small cell lung carcinoma. Thorac Cancer. 2020;11:935–42. 32129931 10.1111/1759-7714.13378 PMC7113046

[8] Gu H Sun L Dou Z. Analysis of lung adenocarcinoma with bone metastasis: a case report. Transl Lung Cancer Res. 2020;9:389–92. 32420080 10.21037/tlcr.2020.03.11 PMC7225164

[9] Higuchi T Sugisawa N Park JH. Osimertinib regressed an EGFR-mutant lung-adenocarcinoma bone-metastasis mouse model and increased long-term survival. Transl Oncol. 2020;13:100826. 32659740 10.1016/j.tranon.2020.100826 PMC7356269

[10] Zhang Y Zhang X Wang F. High quality of 58-month life in lung cancer patient with brain metastases sequentially treated with gefitinib and Osimertinib. Open Med (Wars). 2021;16:1602–7. 34722896 10.1515/med-2021-0379 PMC8546288

[11] Nishii Y Hataji O Ito K. Efficacy of Osimertinib in a patient with non-small cell lung cancer harboring epithelial growth factor receptor exon 19 deletion/T790M mutation, with poor performance status. Mol Clin Oncol. 2018;8:246–9. 29399354 10.3892/mco.2017.1522 PMC5774518

[12] Santini D Barni S Intagliata S. Natural history of non-small-cell lung cancer with bone metastases. Sci Rep. 2015;5:18670. 26690845 10.1038/srep18670 PMC4687045

[13] Tipsmark LS Bünger CE Wang M. Healthcare costs attributable to the treatment of patients with spinal metastases: a cohort study with up to 8 years follow-up. BMC Cancer. 2015;15:354. 25939658 10.1186/s12885-015-1357-z PMC4424566

